# Targeting periosteal SSCs for aged bone defects

**DOI:** 10.18632/aging.102869

**Published:** 2020-02-23

**Authors:** Youngjae Jeong, Dongsu Park

**Affiliations:** 1 Department of Molecular and Human Genetics, Baylor College of Medicine, Houston, TX 77030, USA; 2 Center for Skeletal Biology, Baylor College of Medicine, Houston, TX 77030, USA; 3 Department of Pathology and Immunology, Baylor College of Medicine, Houston, TX 77030, USA

**Keywords:** skeletal stem cells, fracture repair, stem cell migration, aging

Aging is a key risk factor for degenerative bone and cartilage disorders, such as osteoporosis and arthritis. Like other age-associated functional declines, at least some of the defects in bones and cartilage in the elderly have been attributed to changes in the populations and functions of stem cells in skeletal tissues. However, due to the cellular complexity in skeletal tissues and poor characterization of endogenous skeletal stem cells (SSCs), age-associated cellular and molecular changes in skeletal stem cells are not well understood.

Skeletal stem cells (SSCs) have been known to reside within bone marrow and are essential for skeletal development, bone modeling/remodeling, and bone repair. With recent advancement of research in the field, distinct SSC populations have been identified in other skeletal tissues, such as calvaria sutures and the periosteum, suggesting the presence of multiple tissue residential SSCs in adult bone. Thus, adult SSCs can be heterogenous population and potentially possess the different function to contribute to bone maintenance and regeneration throughout lifetime. However, one caveat in SSC field is the lack of selective markers to distinguish a rare subset of SSCs *in vivo*, and hence makes it challenging to identify and characterize various populations of SSCs in different locations.

Many previous studies have focused on the discovery of molecular markers to define and characterize the subset of SSC population. To briefly mention, Nestin-GFP^+^, Leptin Receptor^+^ (LepR^+^), and Mx1^+^ cells are perivascular stromal cells with multilineage differentiation capacity toward mesenchymal lineage, while there are subtle differences in their *in vivo* function and lineage differentiation potential [1–3]. Both Nestin-GFP^+^ and LepR^+^ cells express high levels of hematopoietic stem cell (HSC) niche factors (Cxcl12 and Stem cell factor [Scf]) to play a critical role in HSC maintenance, but they have different *in vivo* lineage differentiation potential [1,2]. Nestin-GFP^+^ cell contribute mainly to osteochondral lineage, whereas LepR^+^ cell are the major source of osteoblast and adipocytes, although LepR^+^ cells also can turn into osteochondogenic cells with bone injury [1,2]. In contrast, Mx1^+^ cells predominantly differentiate into osteoblasts with minimal and no contribution toward the adipocyte and chondrocyte, respectively, under both normal and fractured conditions [3]. Unlike majority of bone marrow mesenchymal stromal cells that are perivascular, Gremlin1^+^ cells mainly reside within the metaphysis regions and they are required for bone development, remodeling, and fracture repair [4]. More recently, calvaria suture and periosteum have been identified as niche for SSCs as well. Prx1 and Axin2 labeled cells in suture are self-renewable and have potential to differentiate into osteoblasts during craniofacial development and injury healing [5,6].

Despite the recent discoveries and improvement in our understanding in SSCs, the functional roles of different SSC subpopulation and their differential responses to bone injury have not been thoroughly studied. Furthermore, whether they have different regulatory mechanisms under the stress or aging are essentially unknown. Our recent data showed that Mx1 and αSMA-GFP combination can selectively label endogenous periosteal SSCs (P-SSCs) that maintain *in vivo* stem cell function [7]. More importantly, these Mx1^+^αSMA-GFP^+^ P-SSCs mainly contributed in replenishing the bone injury repairing osteoblasts *in vivo*, while there were only a few Mx1^+^ or Mx1^+^Nestin-GFP^+^ bone marrow SSCs (BM-SSCs) present at the bone injury site, suggesting that P-SSCs and BM-SSCs have differential role and regulatory mechanism during bone healing following injury [7]^(7)^. Previous studies showed that LepR and Gremlin1 labeled BM-SSCs contribute to bone injury healing [2,4], but these studies have used fracture models which could be difficult to discern the proportional contribution of P-SSCs and BM-SSCs. And yet, it should not be ignored that different results can be obtained depending on the age of animals, different injury model and injury location. BM-SSCs are mainly available for trabecular bone remodeling and HSC maintenances. Thus, BM-SSCs may have greater contribution in trabecular bone injury healing, while the cortical bone injury repair is mainly contributed by P-SSCs. Indeed, the cellular intrinsic differences between P-SSCs and BM-SSCs may exist, where periosteum cells can undergo both endochondral ossification and intramembranous ossification while bone marrow cells can only undergo the latter process [8]. Our recent work showed potential molecular mechanism of injury response of P-SSCs. Mx1^+^αSMA^+^ P-SSCs can respond rapidly to bone injury and recruited to the injury site [7]. Furthermore, these P-SSCs selectively express CCR5, a receptor for CCL5, and exhibited migratory function with improved bone healing upon CCL5 treatment [7]. In supportive of potential role of CCR5/CCL5 in injury healing, Ccr5 or Ccl5-deficient mice exhibited compromised bone healing capacity [7].

In conclusion, our data and those of others support the notion that there is multiple subset of SSCs reside in various tissue location with different regulatory roles under steady-state and stressed condition. In particular, periosteum contains specific subset of SSCs that possess the distinct molecular and cellular signature from BM-SSCs and may have essential role in bone maintenance and repair in aging. Further understanding whether P-SSCs undergo cellular and molecular changes during aging and which regulatory mechanisms control age-associated P-SSC changes promises new approaches to degenerative bone diseases and defects.
